# Prevention of vision loss protects against age-related impairment in learning and memory performance in DBA/2J mice

**DOI:** 10.3389/fnagi.2013.00052

**Published:** 2013-09-18

**Authors:** Aimée A. Wong, Richard E. Brown

**Affiliations:** Department of Psychology and Neuroscience, Dalhousie UniversityHalifax, NS, Canada

**Keywords:** aging, vision, learning, memory, glaucoma, mice, Timoptic-XE

## Abstract

The DBA/2J mouse is a model of pigmentary glaucoma in humans as it shows age-related increases in intraocular pressure (IOP), retinal ganglion cell death and visual impairment. Previously, we showed that visual ability declines from 9 to 12 months of age and visual impairment is correlated with poor learning and memory performance in visuo-spatial tasks but not in tasks that do not depend on visual cues. To test the “sensory impairment” hypothesis of aging, which postulates that sensory impaired individuals are disadvantaged in their performance on psychometric tests as a direct result of difficulties in sensory perception, we treated DBA/2J mice with a conventional glaucoma medication used in humans (Timoptic-XE, 0.00, 0.25, or 0.50%) daily from 9 weeks to 12 months of age to determine whether prevention of vision loss prevented the decline in visuo-spatial learning and memory performance. At all ages tested (3, 6, 9, and 12 months of age), mice treated with Timoptic-XE (0.25 and 0.50%) maintained a high level of performance, while 12 month old control mice (0.00%) exhibited impaired performance in visually-dependent, but not non-visual tasks. These results demonstrate that when sensory function is preserved, cognitive performance is normalized. Thus, as in many aging humans, DBA/2J mice show age-related decrements in performance on visually presented cognitive tests, not because of cognitive impairment but as a direct consequence of poor visual ability. Our results demonstrate that age-related impairment in performance in visuo-spatial tasks in DBA/2J mice can be prevented by the preservation of visual ability.

## Introduction

DBA/2J mice naturally develop a genetically determined form of glaucoma that resembles iris stromal atrophy and iris pigment dispersion syndrome (John et al., 1998; Chang et al., 1999; Anderson et al., 2002), which lead to pigmentary glaucoma in humans (Richter et al., 1986; Mastropasqua et al., 1996; Siddiqui et al., 2003). DBA/2J mice exhibit the hallmarks of human pigmentary glaucoma, notably spontaneous elevated intraocular pressure (IOP), atrophic excavation of the optic nerve head, progressive loss of retinal ganglion cells and visual impairment (John et al., 1998; Schuettauf et al., 2004; Libby et al., 2005a; Saleh et al., 2007; Wong and Brown, 2007).

Previously, we determined that a conventional glaucoma medication used in humans to lower IOP by reducing aqueous humor production, Timolol maleate (Timoptic-XE®), was successful in preventing the behavioral, ocular, and neural symptoms of vision loss exhibited in untreated DBA/2J mice at 12 months of age. Specifically, DBA/2J mice receiving Timoptic-XE treatment from 9 weeks of age maintained a high level of performance in behavioral vision tasks at 12 months of age, while untreated mice exhibited impaired visual performance. Timoptic-XE therapy also reduced IOP and cell loss in the ganglion cell layer of the retina and prevented somal shrinkage and the decrease in the transneural labeling of wheat germ agglutinin conjugated to horseradish peroxidase (WGA-HRP) in the superior colliculus that occurred in untreated mice at 12 months of age (Wong and Brown, 2012).

For the present study, we evaluated measures of learning and memory in visuo-spatial and olfactory tasks in order to determine the relationship between vision and cognitive function in aged mice. Age-related decline in visual ability in humans has been associated with a decline in recall memory but not verbal ability, while an age-related decline in hearing ability was not associated with a decline in any cognitive domain (Anstey et al., 2003, 2001b). Age-related visual but not hearing impairment has been associated with poorer scores in memory and cognitive speed in adults aged 85 years and older (Gussekloo et al., 2005), low cognitive function and cognitive decline in older Mexican Americans (Reyes-Ortiz et al., 2005) and in women over 69 years of age (Lin et al., 2004).

Since many of the tests used to evaluate cognitive ability in humans are visually-dependent, it is not surprising that poorer scores in these cognitive tasks are associated with visual impairment. Specifically, visual impairment has been associated with poorer scores in memory and cognition on the Mini-Mental State Exam, which has both visual and auditory components (Lin et al., 2004; Gussekloo et al., 2005; Reyes-Ortiz et al., 2005; Ishil et al., 2008) and poorer performance in visually-presented tests of memory, such as the symbol recall, picture recall and The 12-Word Learning Tests (Anstey et al., 2001b, 2003; Gussekloo et al., 2005); verbal memory on The Visual Verbal Learning Test (Valentijn et al., 2005); cognitive flexibility on The Concept Shifting Task (Valentijn et al., 2005); information processing speed and attention on the Color Word Stroop and Digit Symbol Substitution tests (van Boxtel et al., 2001; Anstey et al., 2001b, 2003; Gussekloo et al., 2005; Valentijn et al., 2005); memory span on WMS®-III Spatial Span forward test (Clay et al., 2009); and fluid intelligence on the Mini-Mental State Exam and WASI Matrix Reasoning test (Clay et al., 2009).

Four theories have been proposed to account for the high correlation between the decline of sensory and cognitive abilities in humans (Li and Lindenberger, 2002; Valentijn et al., 2005). The “common cause” hypothesis suggests that the decline of sensory and cognitive functioning in old age is caused by a common mechanism that results in widespread neuronal atrophy, reducing all aspects of central nervous system functioning. The “sensory deprivation” hypothesis proposes that prolonged reductions in the quality or quantity of sensory input leads to cognitive deterioration due to neuronal atrophy. The “resource allocation” hypothesis states that sensory-impaired individuals must allocate more attentional resources to perceive and interpret sensory information and, as a consequence, there are fewer resources left for attending to cognitively demanding tasks. However, the finding that there is a strong relationship between visual but not hearing impairment and cognitive decline, in combination with the observation that cognitive ability is typically measured using visually-presented tests has prompted the formulation of the “sensory impairment” hypothesis, which postulates that sensory impaired individuals are disadvantaged in their performance on psychometric tests as a direct result of difficulties in sensory perception (Lindenberger et al., 2001; Gussekloo et al., 2005; Valentijn et al., 2005; van Boxtel et al., 2001). Thus, visually impaired individuals perform poorly on visually presented cognitive tests, not because of cognitive impairment but as a direct consequence of poor visual ability. Furthermore, the “sensory impairment” theory hypothesizes that visual and cognitive ability in visually impaired individuals can be dissociated by assessing cognitive functioning with tests that are not dependent on vision, such as auditory cognitive tests (Gussekloo et al., 2005) and that treatment of the impaired sensory function should result in improvement of cognitive performance (Valentijn et al., 2005).

Thus, the “sensory impairment” theory of age-related cognitive dysfunction can be evaluated by testing three hypotheses: (1) visually impaired individuals should perform poorly on visually presented cognitive tasks, (2) visual ability in visually impaired individuals should be dissociated from cognitive ability when cognitive functioning is assessed using tests that are not dependent on vision, and (3) improvement of impaired visual function should result in improvement of cognitive performance. We tested these three hypotheses using the DBA/2J mouse model of pigmentary glaucoma by evaluating the effect of Timoptic-XE on performance in two behavioral tasks that measure learning and memory: the Morris water maze, which is dependent on the detection of visual cues and the conditioned odor preference task, which does not rely on visual ability. By measuring cognitive performance on tasks that depend on two different sensory modalities (vision and olfaction), we can test the hypotheses that age-related visual impairment (1) reduces cognitive performance on visual-spatial learning in the Morris water maze but (2) not in the Pavlovian conditioned odor preference task, and (3) that improvement of visual function with Timoptic-XE would prevent the impaired cognitive performance in visual spatial learning and memory.

## Methods

Mice were treated in accordance with the regulations set forth by the Canadian Council on Animal Care and the experimental protocol was approved by the Dalhousie University Committee on Animal Care (Protocol# 05-134 and 08-094). DBA/2J mice (JAX stock #000671) obtained from The Jackson Laboratory (Bar Harbor, ME) at 5 weeks of age were used in this experiment. Mice were housed in same-sex pairs in clear plastic cages (29.2 × 18.4 × 12.7 cm) with metal wire lids and fed Purina rodent chow (#5001) and tap water *ad libitum*. The colony room was maintained at a temperature of 22 ± 2°C, with a 12:12 h reversed light:dark cycle (lights off at 9:45 am).

Mice were divided into three experimental groups and given Timoptic-XE (Merck Frosst Canada Ltd, Quebec, CA), a sterile ophthalmic gel-forming solution which contained (1) 6.8 mg of timolol maleate (0.50% Timoptic-XE), (2) 3.4 mg of timolol maleate (0.25% Timoptic-XE) or (3) 0.00% Timoptic-XE (control) throughout the experimental period. The controls were given an aqueous solution containing 0.6% Gelrite gellan gum (Sigma-Aldrich Canada Ltd.; Oakville, ON), the inactive ingredient in Timoptic-XE that causes it to form a gel (Carlfors et al., 1998).

Mice were given one drop of solution in each eye daily, from 9 weeks to 12 months of age. To prevent mice from grooming away the eye drop, they were given one “Fruity O's” cereal treat (President's Choice, Brampton, ON) to eat immediately after the eye drop procedure. Mice were given the behavioral test battery, which included the visual water task, Morris water maze and the conditioned odor preference task, at 3, 6, 9, and 12 months of age. All behavioral testing was completed during the dark (active) portion of the light: dark cycle. Table 1 shows the number of male and female mice in each experimental group that completed behavioral testing at each age. Following behavioral testing at each age, 12 mice (4 mice from each drug group) were sacrificed for histological evaluation of retinal and neural parameters as reported by Wong and Brown (2012).

**Table 1:** **Number of male (M) and female (F) mice of each drug group that completed behavioral testing at 3, 6, 9, and 12 months of age**.

**Drug group**	**3 months**	**6 months**	**9 months**	**12 months**
0.50% Timoptic-XE	4 M, 4 F	2 M, 2 F	3 M, 4 F	2 M, 1 F
0.25% Timoptic-XE	4 M, 4 F	2 M, 1 F	4 M, 2 F	1 M, 2 F
0.00% Timoptic-XE (0.60% Gelrite)	4 M, 4F	2 M, 2 F	4 M, 4 F	2 M, 1 F
Total	12 M, 12 F	6 M, 5 F	11 M, 10 F	5 M, 4 F

### Visual water task

In the visual water task, mice were trained to associate a visual stimulus with escape from water (Prusky et al., 2000; Wong and Brown, 2006). Briefly, mice were trained in a trapezoidal-shaped pool to discriminate between visual stimuli, which were sinusoidal gratings (S+), formed by gradual sine-wave variations in luminance and a homogeneous gray screen (S−) (LabVIEW, National Instruments, Vaudreuil-Dorion, QC), which were presented on two computer monitors as depicted in Wong and Brown (2006, 2012). A clear Plexiglas platform providing escape from water, was placed in front of the computer screen displaying the positive visual stimulus (S+) and was situated 1 cm below the surface of the water, rendering it invisible to the observer. No platform was placed in front of the computer monitor displaying the negative visual stimulus (S−). Visual water task training/testing was conducted over 25 consecutive days starting with pretraining (1 day, 6 trials), visual detection (8 days, 8 trials/day), pattern discrimination (8 days/8 trials/day) and visual acuity testing (8 days, 8 trials/day) as described in detail in Wong and Brown (2006, 2012).

#### Statistical analyses

One-way ANOVAs were used to analyze drug group differences in the percentage of correct responses on Day 8 for the visual detection and pattern discrimination tasks and the visual acuity threshold at each age. *Post-hoc* analyses were conducted using Fisher's PLSD tests. All statistical analyses were performed using Statview 5.0, Abacus Concepts, Inc (Berkeley, CA).

### The morris water maze

In the Morris water maze (Morris, 1981), mice are required to learn the location of a platform that is hidden in a pool of opaque water by using distal visual stimuli located around the testing room (Brown and Wong, 2007; Wong and Brown, 2007). Mice were tested in four phases over eight consecutive days: acquisition, reversal, probe and visible platform tests. During acquisition, mice were trained over 3 days (4 trials/day) to find a hidden escape platform that was located in the Northeast quadrant. The mice had a maximum of 60 s to find the platform. If they were unsuccessful, they were gently guided to the platform and required to stay on the platform for ~15 s before being removed and placed back into their home cage. Latency and swim distance to find the platform were recorded and daily means (4 trials) served as measures of learning. For reversal training the hidden platform was moved to the opposite quadrant of the pool (Southwest) and mice were required to learn the new location over 3 days (4 trials/day). On the 7th day, the platform was removed from the pool and spatial memory was evaluated in a probe trial. The amount of time spent swimming in each quadrant of the pool and the number of annulus crossings were used as measures of memory. The visible platform phase (1 day, 4 trials) occurred on the last day of testing and served as a test for visual deficits. The platform was placed in the Northwest quadrant and was made visible by a colorful flag and red top that extended above the surface of the water. Latency and swim distance to find the visible platform for each trial were used as measures of visual ability.

#### Statistical analyses

Sex differences were analyzed for all measures evaluated in the Morris water maze but because there were no significant main effects or interactions with sex, data for male and female mice in each drug group were pooled. Differences in latency and swim distance to find the platform during acquisition and reversal phases were analyzed using 3 × 6 (drug group × days) ANOVAs. Drug effects at each age were analyzed using one-way ANOVAs. *Post-hoc* analyses were conducted using Fisher's PLSD tests for all analyses (Statview 5.0, Abacus Concepts, Inc; Berkeley, CA).

### The conditioned odour preference task

The conditioned odor preference task, developed by Schellinck, Forestell and Lolordo (2001), was used to test olfactory-based learning and memory. It was conducted in two phases: training and testing (Brown and Wong, 2007; Wong and Brown, 2007). The training apparatus consisted of a standard clear, plastic, housing cage (29.2 × 18.4 × 12.7 cm) and the testing apparatus was a clear acrylic three compartment box (69 × 20 × 20 cm) with removable acrylic doors in the dividing walls that allowed the mouse to move between compartments. The floors of the training and testing apparatus were covered with Pro-Chip bedding (PWI Industries Inc, Saint Hyacinthe, QC) to a depth of ~2.5 cm.

Lemon (Linalool) and rose (phenyl acetate) odors were obtained from Sigma-Aldrich Canada Ltd. (Oakville, ON) and were diluted to a concentration of 15% by mixing 3 ml of odor with 17 ml of 1, 2-propanediol (Caledon Laboratories Ltd; Georgetown, ON). All other odor stimuli, consisted of artificial flavor extracts (Clubhouse; London, ON). Odors were presented in odor pots, which consisted of 0.05 ml of odor placed on a piece of filter paper and covered with a plastic lid that contained 10–12 small holes to allow the odor to escape, and a plastic cup cut to a height of ~1.5 cm. Sugar reward was only given during the training phase and was placed on top of the odor pot containing the positive conditioned odor stimulus (CS+). Table 2 shows the four pairs of odor stimuli that were used in this task and the age when mice were tested for these odor discriminations.

**Table 2:** **The odor discrimination pairs that were used for short-term and long-term memory tests at 3, 6, 9, and 12 months of age**.

**Age (months)**	**Short-term memory test**	**Long term memory test(s)**
3	Rose vs. Lemon	—————————–
6	Almond vs. Banana	1. Rose vs. Lemon (3 months)
9	Cinnamon vs. Coconut	1. Almond vs. Banana (3 months)
		2. Rose vs. Lemon (6 months)
12	Rum vs. Maple	1. Cinnamon vs. Coconut (3 months)
		2. Almond vs. Banana (6 months)
		3. Rose vs. Lemon (9 months)

Long-term memory tests evaluated memory 3, 6, or 9 months after training. Tests were given in the order shown.

Prior to the training phase, mice were food deprived for 22 h/day for up to 4 days until they reached 85–90% of their *ad libitum* weight. They were then randomly assigned to one of two odor training groups. Mice in the X+/Y− group had sugar reward paired with odor X and no sugar with odor Y and mice in the Y+/X− group had sugar reward with odor Y and none with odor X. Mice were trained over 4 days (4 trials/day) to discriminate between odors X and Y. A single odor pot containing either a CS+ (odor X+ or Y+) or a CS− (odor Y− or X−) was buried beneath the Pro-Chip bedding in the center of the training apparatus. The CS+ odor had several pieces of sugar placed on the top and mixed in with the Pro-chip bedding, requiring animals to dig to find them. The CS− odor pot contained no sugar. A trial consisted of removing the mouse from its home cage and placing it in the CS+ or CS− training apparatus for 10 min. The mouse was then returned to its home cage, moved to a odor neutral room and required to wait until the bedding and odor pot were changed for the next training trial (~10 min). On each training day, mice received two CS+ and two CS− trials, with the order of these trials randomized across days.

The memory test occurred on Day 5, 24 h after the last training trial and consisted of habituation (2 min) and testing (3 min) phases. During habituation, pots containing Pro-chip bedding but no odor were placed in the end compartments of the testing apparatus and the mouse was allowed to move between the three compartments for 2 min. The amount of time spent in each of the end compartments was recorded to determine if the mouse exhibited a position preference. Following the habituation trial, the mouse was placed back into its home cage while the apparatus was prepared for the testing trial (~5 min). After being emptied, washed, filled with Pro-chip bedding and rotated 180° to prevent the use of external visual cues, an odor pot containing the training odors (X or Y) was buried in the bedding in each end compartment. No sugar reward was placed in either odor pot for the testing trial. The mouse was placed in the middle compartment, the acrylic doors were opened and the mouse was observed for 3 min. The amount of time spent digging with its forepaws or nose in each odor pot was recorded and the percent of the time spent digging in the CS+ was calculated using the formula: 100 × (time spent digging in the CS+)/[(time spent digging in the CS+) + (time spent digging in the CS−)].

At 6, 9, and 12 months of age, mice were tested for long-term memory of previously learned odor discrimination(s) before being trained and tested for a new odor discrimination. Long-term memory tests were conducted before the training phase for the new odor discrimination and followed the procedure of the testing phase described above. In the case of multiple long-term odor memory tests (9 and 12 months of age), only one memory test was given per day, with the most recently learned discrimination presented first, as shown in Table 2.

#### Statistical analyses

Sex differences in the percentage of time digging in the CS+ were analyzed using a one way ANOVA and because there were no significant main effects of sex, data for male and female mice in each drug group were pooled. Drug group differences at each age and age effects within each drug group in the percentage of time spent digging in the CS+ odor pot were analyzed using one-way ANOVAs with Fisher's PLSD *post-hoc* tests (Statview 5.0, Abacus Concepts, Inc; Berkeley, CA).

## Results

The complete behavioral results for the Visual Water Task are presented in Wong and Brown (2012). There were no sex differences in visual detection, pattern discrimination or visual acuity measures. For the purpose of correlational analyses in this study, the maximal (Day 8) performance in the visual detection and pattern discrimination tasks and maximal visual acuity thresholds were analyzed.

### Visual detection and pattern discrimination

There were no significant differences between drug groups in the percentage of correct responses on Day 8 of visual detection or pattern discrimination testing at 3, 6, or 9 months of age (all *p* > 0.05) but at 12 months of age, mice receiving 0.00% Timoptic-XE had a significantly lower percentage of correct responses in the visual detection task [*F*_(2, 7)_ = 33.512, *p* = 0.0003] than mice receiving 0.25 and 0.50% Timoptic-XE (*p* = 0.0016 and *p* < 0.0001, respectively) (Table 3A). Likewise, in the pattern discrimination task at 12 months of age, mice receiving 0.00% Timoptic-XE had significantly fewer correct responses [*F*_(2, 7)_ = 9.165, *p* = 0.0111] than 0.25 and 0.50% Timoptic-XE groups (*p* = 0.01231 and *p* = 0.0063, respectively) (Table 3B).

**Table 3:** **Mean (±*S.E.*) percent correct on Day 8 of the visual detection (VD 8) and pattern discrimination task (PD 8) and visual acuity thresholds (c/deg) for each drug group in the Visual Water task at 3, 6, 9, and 12 months of age**.

**Drug group**	**3 months**	**6 months**	**9 months**	**12 months**
**(A) VD 8 (% CORRECT)**				
0.00% Timoptic-XE	90.625 ± 2.909	87.50 ± 4.694	89.063 ± 5.994	50.00± 0.00
0.25% Timoptic-XE	91.964± 2.813	100.00± 0.00	97.917± 2.083	70.833± 4.167^**^
0.50% Timoptic-XE	93.33± 2.399	100.00± 0.00	91.071± 7.068	83.333± 4.167^***^
**(B) PD 8 (% CORRECT)**				
0.00% Timoptic-XE	87.50± 3.953	90.385± 3.511	78.125± 8.76	46.875± 7.864
0.25% Timoptic-XE	90.179± 2.679	95.313± 4.688	87.50± 4.564	75.00± 0.00^*^
0.50% Timoptic-XE	92.50± 2.673	98.864± 1.136	85.714± 6.916	83.333± 4.167^**^
**(C) VA THRESHOLD (c/deg)**				
0.00% Timoptic-XE	0.403± 0.017	0.363± 0.028	0.441± 0.043	0.00 ± 0.00
0.25% Timoptic-XE	0.499± 0.022	0.448± 0.046	0.427± 0.036	0.433± 0.063^***^
0.50% Timoptic-XE	0.476± 0.023	0.467± 0.031	0.464± 0.035	0.533± 0.027^***^

* Differs from 0.00% at p < 0.05,

** p < 0.01,

*** p < 0.001.

### Visual acuity threshold

Visual acuity threshold was determined for each drug group when the percentage of correct responses dropped below 70% correct. There were no significant differences between drug groups in visual acuity threshold at 3, 6, or 9 months of age (all *P* >0.05). At 12 months of age there was a significant difference between groups [*F*_(2, 7)_ = 71.112, *p* < 0.0001], as mice receiving 0.00% Timoptic-XE had a significantly lower visual acuity than mice receiving 0.25% (*p* < 0.0001) and 0.50% (*p* < 0.0001) Timoptic-XE. In fact, 12 month old 0.00% Timoptic-XE mice did not achieve 70% at any of the spatial frequencies tested and therefore their visual acuity threshold was effectively 0 c/deg (Table 3C).

#### Morris water maze

**Figure 1:**
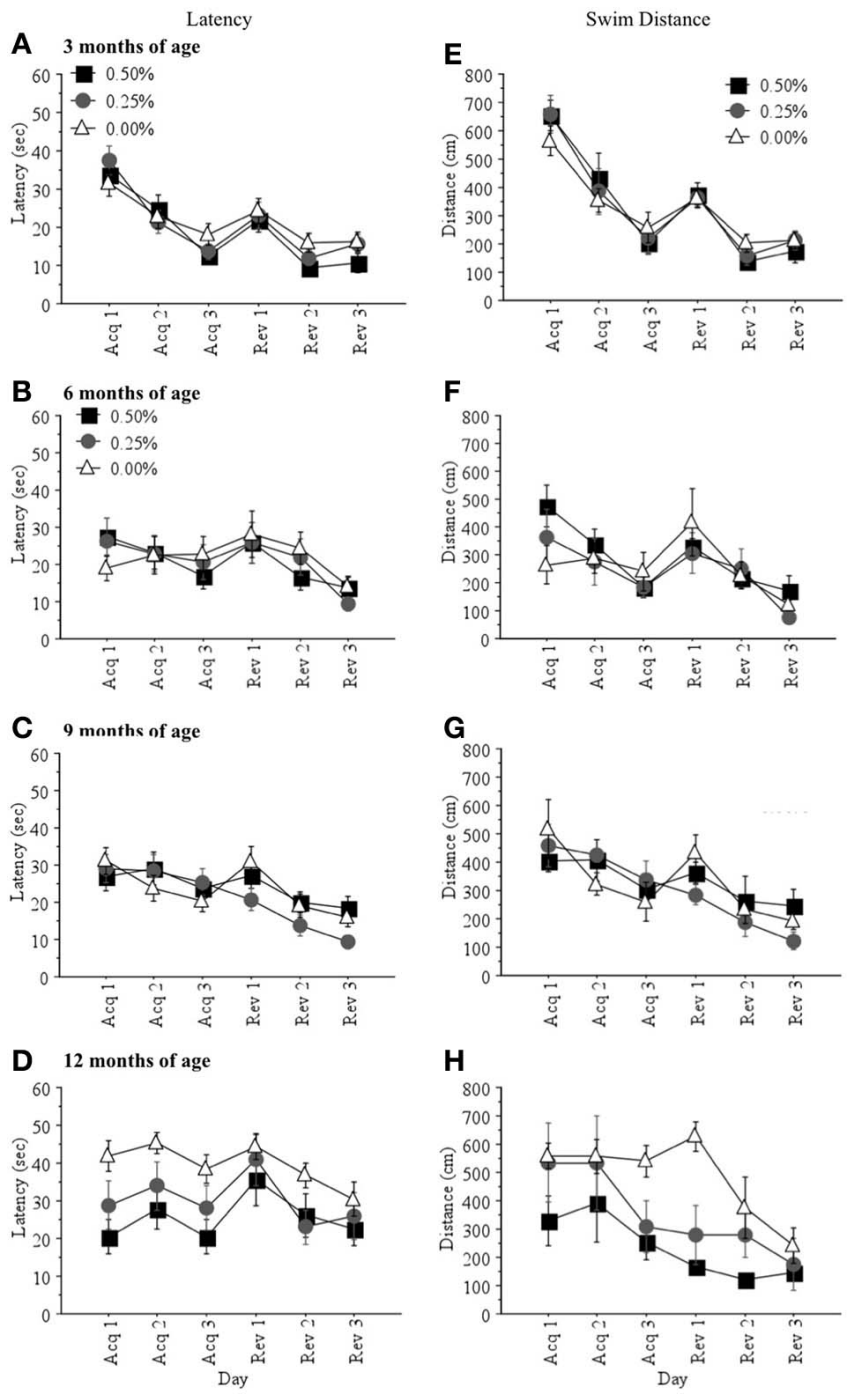
**Mean (±S.E.M.) latency (sec) and swim distance (cm) to find the hidden platform during acquisition and reversal training in the Morris water maze for mice receiving 0.50, 0.25, and 0.00% Timoptic-XE at 3 (A,E), 6 (B,F), 9 (C,G), and 12 (D,H) months of age**.

***Learning Scores***. There were no significant drug group differences in latency (sec) or swim distance (cm) to find the hidden platform during acquisition or reversal training when mice were 3, 6, or 9 months of age (all *p* > 0.05; Figures 1A–C,E–G). However, at 12 months of age, mice receiving 0.00% Timoptic-XE took significantly longer [*F*_(2, 7)_ = 5.727, *p* = 0.0336; Figure 1D] and swam a greater distance [*F*_(2, 7)_ = 7.041, *p* = 0.0211; Figure 1H] to find the hidden platform than mice receiving 0.50% Timoptic-XE (*p* = 0.0137 and *p* = 0.0072, respectively). There were no significant differences in mean swim speed at any age between treatment groups (all *p* > 0.05) that could account for these differences in learning scores at 12 months of age.

### Memory scores

**Figure 2:**
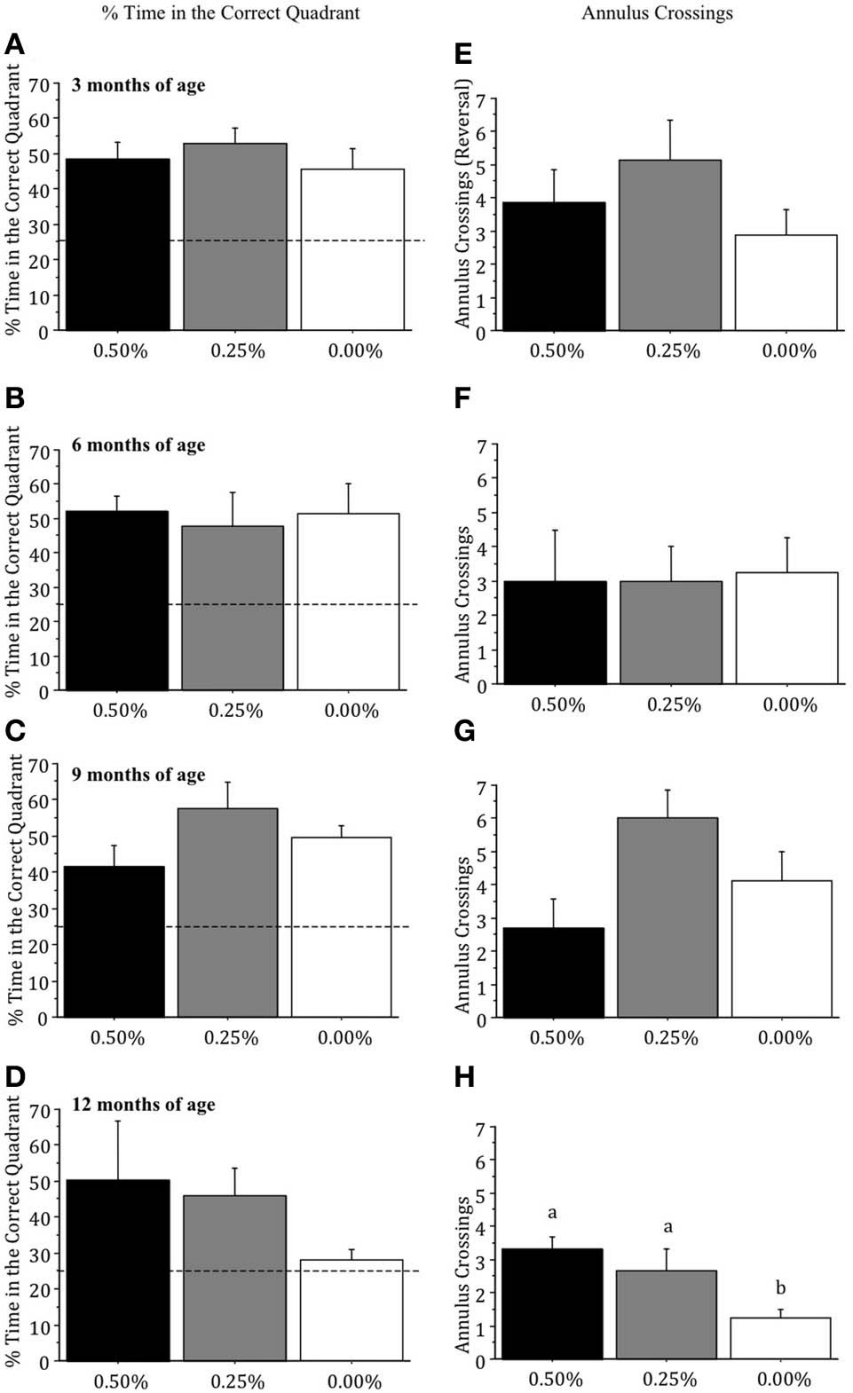
**Mean (±S.E.M.) percentage of time spent in the correct (quadrant) and number of annulus crossings in the probe trial in the Morris water maze, with 25% indicating chance, for mice receiving 0.50, 0.25, and 0.00% Timoptic-XE at 3 (A,E), 6 (B,F), 9 (C,G), and 12 (D, H) months of age.** Dotted line = 25% (chance). Different letters indicate significant differences between groups (*p* < 0.05).

Differences between drug groups in the percentage of time spent in the correct quadrant were not significant when mice were 3, 6, or 9 months of age (all *p* > 0.05; Figures 2A–C). At 12 months of age, the 0.00% Timoptic-XE group spent less time in the correct quadrant than the 0.50 and 0.25% groups (Figure 2D) but this difference failed to reach significance [*F*_(2, 7)_ = 1.745, *p* > 0.05]. When age-related changes were measured within each treatment group, however, the 0.00% Timoptic-XE group showed significantly less time in the correct quadrant at 12 months of age than at 3, 6, or 9 months of age (*p* < 0.05) while the 0.50 and 0.25% Timoptic-XE groups showed no age-related decline in percent time in the correct quadrant.

Annulus crossings during the probe test were not significantly different between drug groups at 3, 6, or 9 months of age (all *p* > 0.05; Figures 2E–G) but at 12 months of age there was a significant difference [*F*_(2, 7)_ = 6.871, *p* = 0.0223], as the 0.00% Timoptic-XE mice had fewer annulus crossings than 0.50% (*p* = 0.0091) and 0.25% Timoptic-XE mice (*p* = 0.0455) (Figure 2H).

### Visible platform test

**Figure 3:**
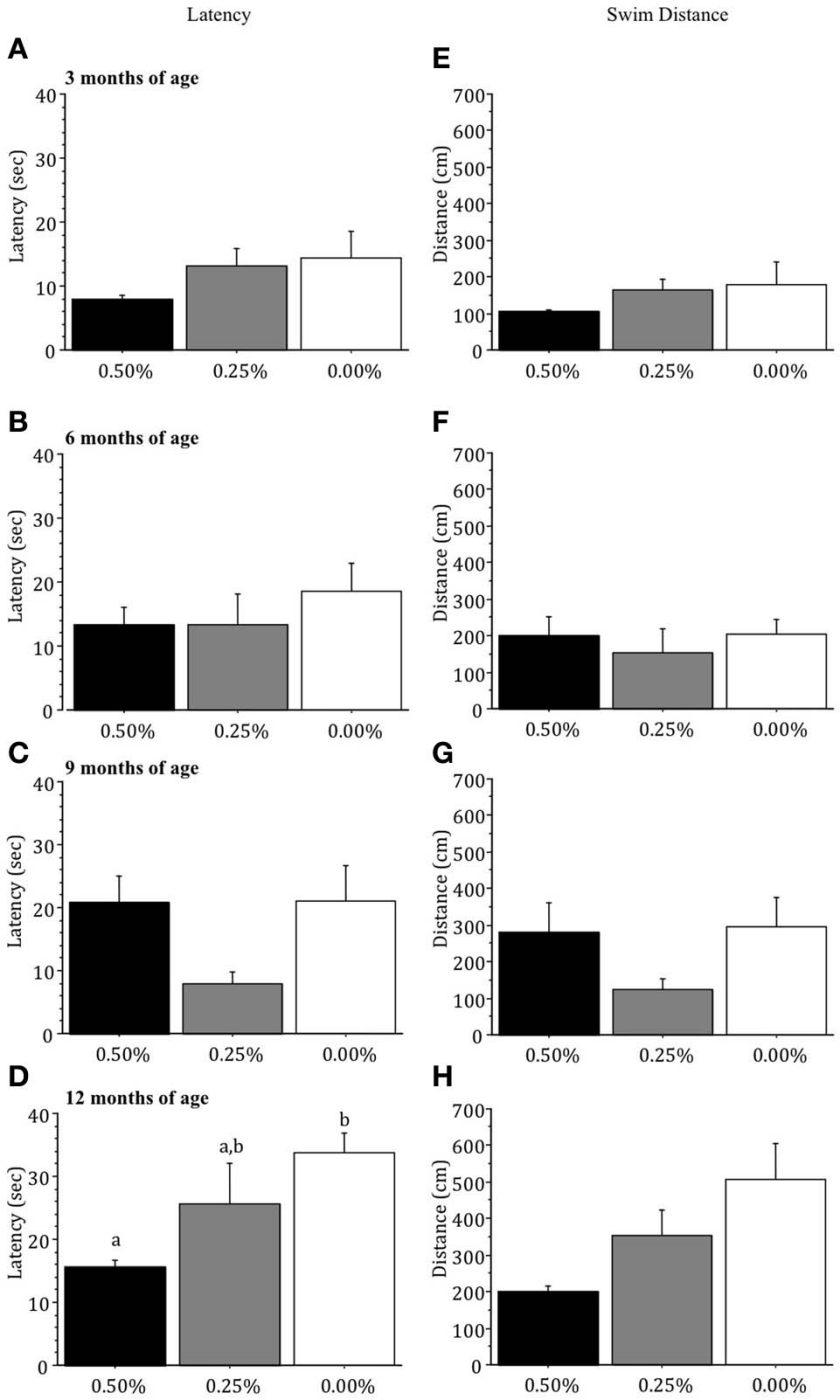
**Mean (±S.E.M.) latency (sec) and swim distance (cm) to find the visible platform in the Morris water maze for mice receiving 0.50, 0.25, and 0.00% Timoptic-XE at 3 (A,E), 6 (B,F), 9 (C,G), and 12 (D,H) months of age.** Different letters indicate significant differences between groups (*p* < 0.05).

Drug groups did not differ significantly in the latency (sec) or swim distance (cm) to find the visible platform at 3, 6, or 9 months of age (all *p* > 0.05; Figures 3A–C,E–G) but at 12 months of age mice receiving 0.00% Timoptic-XE took significantly longer to reach the visible platform [*F*_(2, 7)_ = 5.316, *p* = 0.0394; Figure 3D] than 0.50% (*p* = 0.0139) Timoptic-XE mice but did not swim a greater distance (Figure 3H).

#### Conditioned odor preference task

**Figure 4:**
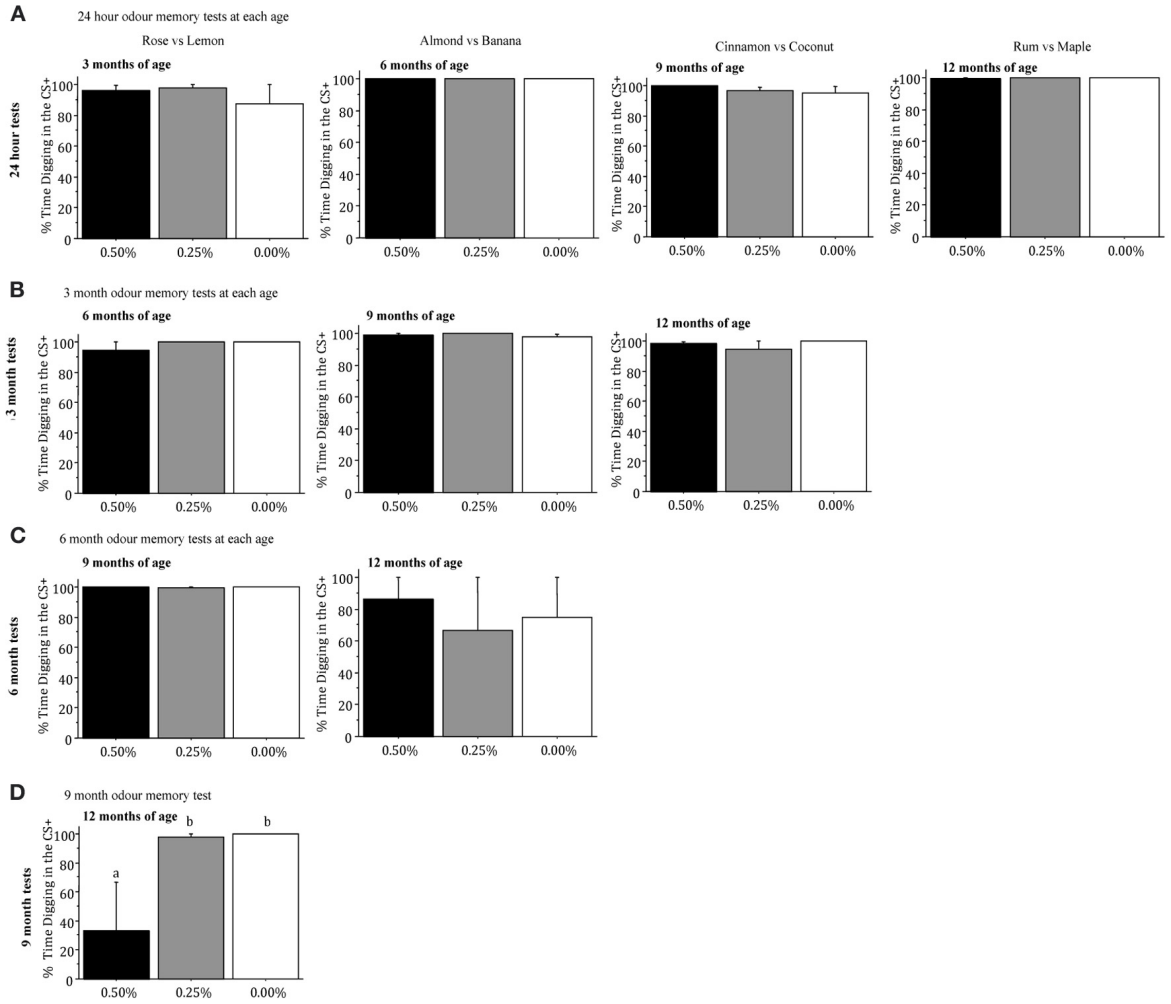
**Mean (±S.E.M.) percentage of time spent digging in the CS+ when mice were tested for memory 24 h (A), 3 months (B), 6 months (C), and 9 months (D) after training in the conditioned odor preference task for mice receiving 0.50, 0.25, and 0.00% Timoptic-XE.** Different letters indicate significant differences between groups (*p* < 0.05).

***Percentage of Digging in the CS+***. The three drug groups did not differ significantly in the percentage of digging in the CS+ in the conditioned odor preference task when they were tested for memory of the rose and lemon odors (the first discrimination) 1 day, 3 months, or 6 months after training (all *p* > 0.05; Figures 4A–C). However, 9 months after mice had received training for the rose vs. lemon discrimination, when they were 12 months of age, there were significant drug group differences [*F*_(2, 7)_ = 4.743, *p* = 0.0499], as mice receiving 0.50% Timoptic-XE performed significantly worse than mice receiving 0.25% (*p* = 0.0380) or 0.00% Timoptic-XE mice (*p* = 0.0257) (Figure 4D).

When mice were taught a second odor discrimination (almond vs. banana) at 6 months of age, there were no significant differences in the percentage of digging in the CS+ when mice were tested for memory 1 day, 3 months, or 6 months after training (all *p* > 0.05; Figures 4A–C]. Drug groups did not differ significantly in the percentage of digging in the CS+ when mice were tested for memory of a third odor discrimination pair (cinnamon vs. coconut) 24 h after training at 9 months of age or 3 months later (all *p* > 0.05, Figures 4A,B). Finally, there were no significant differences between drug groups in the percentage of digging in the CS+ in the memory test that was conducted 1 day after mice had finished training of a fourth odor discrimination pair (rum and maple) at 12 months of age [*F*_(2, 7)_ = 1.225, NS; Figure 4A].

Inspection of Figure 4 shows that in all of the 24 h and 3 month memory tests mice in all groups spent >90% of their time digging in the CS+ (chance = 50%). In the 6 month tests there was virtually 100% digging in rose vs. lemon odors but much less digging in all groups given almond vs. banana odors. Finally, in the 9 month test, mice in the 0.00 and 0.25% Timoptic-XE groups had almost 100% digging in the CS+ while mice receiving 0.50% Timoptic-XE were at chance. This indicates that mice had a reliable memory for conditioned odor preferences for up to 9 months.

#### Correlations between behavioral measures

Pearson product moment correlations (Statview 5.0; Abacus Concepts, Inc., Berkeley, CA) were used to analyze the relationship between behavioral measures of visual ability, learning and memory in the Morris water maze and memory in the conditioned odor preference task. The correlation matrix between behavioral measures using mice from all four ages (*N* = 38) is shown in the top triangle in Table 4. Only the data from the age at which the mouse was sacrificed was used for this analysis (one measurement per mouse). A separate correlation matrix for 12 month old mice (*N* = 9) is shown in the bottom triangle of Table 4.

**Table 4:** **Pearson product moment correlations showing the relationship between measures of vision and learning and memory behaviour in all mice tested (*N* = 38 unless otherwise stated, top triangle) and mice tested at 12 months of age only (*N* = 9, bottom triangle)**.

	**% correct VD 8**	**% correct PD 8**	**VA threshold**	**MWM latency**	**MWM distance**	**% time quad**	**Annulus crossings**	**Visible latency**	**Visible distance**	**Rose vs. lemon**	**Almd vs. banana**	**Cinn vs. coco**
% correct VD 8		0.779^***^	0.533^***^	−0.614^***^	−0.488^**^	0.330^*^	0.312	−0.657^***^	−0.736^***^	−0.034	0.132 *N* = 27	0.146 *N* = 17
% correct PD 8	0.848^**^		484^**^	−0.568^***^	−0.551^***^	0.226	0.187	−0.565^***^	−0.688^***^	−0.041	0.242 *N* = 27	0.161 *N* = 17
Visual acuity threshold	0.913^***^	0.891^**^		−0.441^**^	−0.566^***^	0.321^*^	0.144	−0.555^***^	−0.615^***^	−0.203	0.280 *N* = 27	0.116 *N* = 17
MWM latency (sec)	−0.829^**^	−0.561	−0.701^*^		0.584^***^	−0.124	−0.109	0.667^***^	0.619^***^	−0.118	−0.194 *N* = 27	−0.291 *N* = 17
MWM distance (cm)	−0.876^**^	−0.921^***^	−0.926^***^	0.603		−0.330^*^	−0.137	0.476^**^	0.554^***^	0.277	−0.160 *N* = 27	−0.149 *N* = 17
% time quadrant	0.382	0.440	0.667^*^	−0.310	−0.550		0.563^***^	−0.217	−0.301	0.173	0.173 *N* = 27	−0.249 *N* = 17
Annulus crossings	0.736^*^	0.574	0.872^**^	−0.623	−0.720^*^	799^**^		−0.130	−0.191	0.013	0.241 *N* = 27	−0.526* N = 17
Visible latency (sec)	−0.691^*^	−0.833^**^	−0.753^*^	0.316	0.831^**^	−0.491	−0.737^*^		0.922^***^	0.076	−0.281 *N* = 27	−0.583^***^ N = 17
Visible distance (cm)	−0.772^*^	−0.688^**^	−0.800^**^	0.511	917^***^	−0.301	−0.652	0.884^**^		0.063	−0.337 *N* = 27	−0.318 *N* = 17
Rose vs. lemon	−0.731^*^	−0.477	−0.489	0.442	0.532	0.118	−0.307	0.579	0.494		−0.114 *N* = 27	0.055 *N* = 17
Almond vs. banana	0.386	0.024	0.202	−0.621	−0.207	0.059	0.319	−0.290	−0.319	−0.362		−0.166 *N* = 17
Cinnamon vs. coconut	−0.307	−0.314	−0.191	0.632	0.141	0.023	0.060	−334	0.051	0.041	−0.283	
Rum vs. maple	−0.169	−0.187	−0.353	0.201	0.360	−0.718^*^	−0.516	0.287	0.336	−0.197	0.118	−0.081

* p < 0.05,

** p < 0.01,

*** p < 0.001.

The percentage of correct responses in the visual detection task on Day 8 was significantly correlated with Day 8 performance in the pattern discrimination task and visual acuity threshold. Pattern discrimination performance and visual acuity threshold were also significantly correlated when using the data from mice at all ages and from only mice at 12 months of age (Table 4).

**Figure 5:**
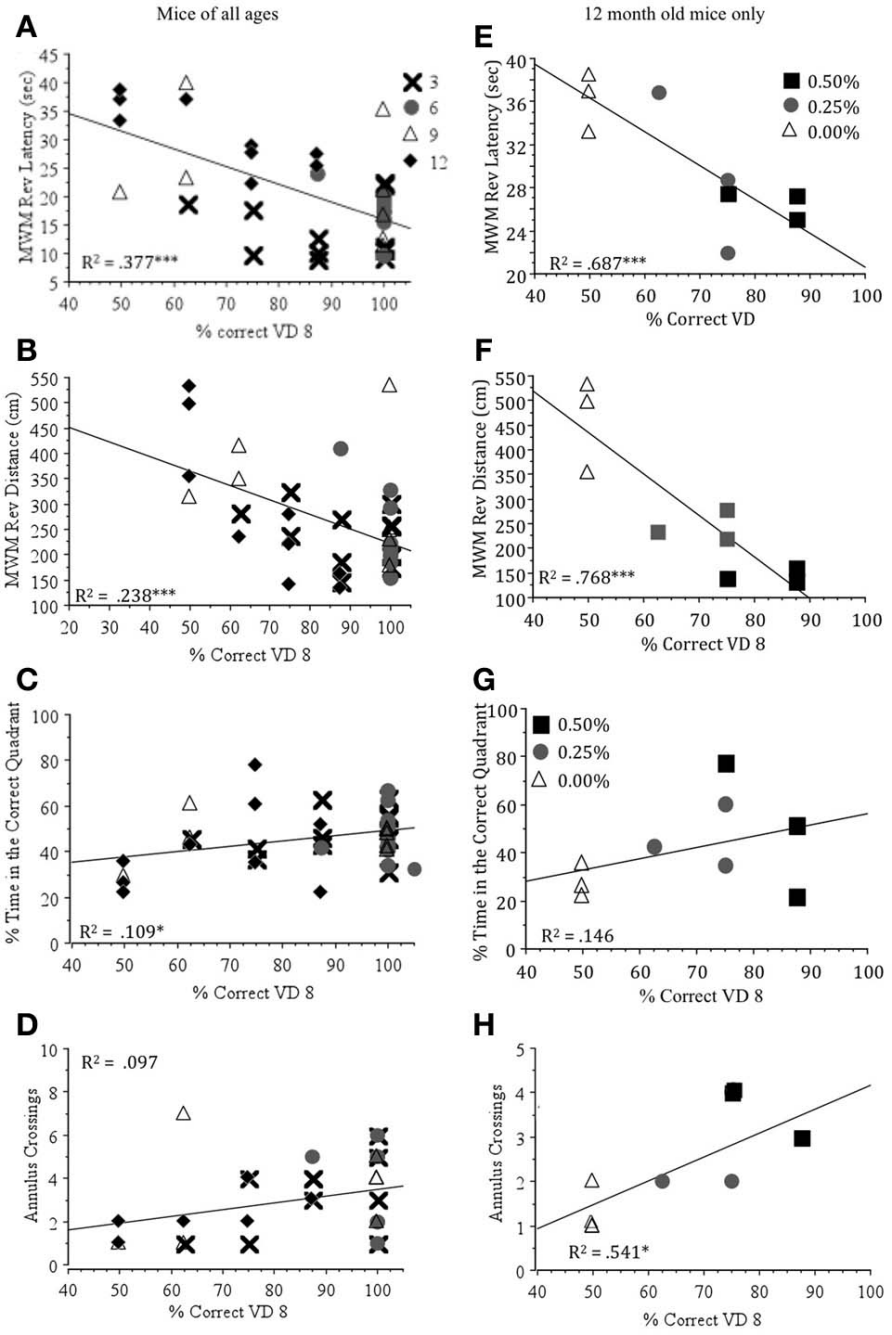
**Regression plots showing the correlation between the percent correct on Day 8 of the visual detection task (% Correct VD 8) and latency (sec) (A,E), swim distance (cm) (B,F), the percentage of time spent in the correct quadrant (C,G) and annulus crossings (D,H) in the probe trial in Morris water maze for mice at all ages (*N* = 38) and at 12 months of age (*N* = 9).** ^*^p < 0.05, ^***^p < 0.01.

Visual ability was significantly negatively correlated with both measures of learning to find the hidden platform in the Morris water maze (latency and distance during reversal days) for all mice and 12 month old mice (Table 4). On the other hand, measures of visual ability had positive, but less significant correlations with measures of memory (% time in the correct quadrant and annulus crossings). When depicted graphically (Figure 5), it is clear that increased visual ability results in shorter latencies and swim distance in the learning trials and greater time in the correct quadrant and more frequent annulus crossings in the memory trials of the Morris water maze. Latency to locate the visual platform was significantly correlated with visual ability indicating that this is a useful crude measure of visual ability.

Visual ability did not predict performance in the conditioned odor preference task at any age (Table 4), although at 12 months of age most correlations were negative, indicating that mice with poorer vision spent more time digging in the CS+ than those with good vision, a finding that we have reported previously (Wong and Brown, 2007).

## Discussion

Our results showed that Timoptic-XE (0.50 and 0.25%) prevented the age-related decline of visual ability demonstrated in control mice (0.00% Timoptic-XE) at 12 months of age (Wong and Brown, 2012). The visual deficits exhibited by control mice were significantly correlated with age-related impairments in visuo-spatial learning and memory performance in the Morris water maze when mice of all ages were included in the analysis and in only 12 month old mice (Table 4). Visual detection performance accounted for ~70% of the variability in latency and swim distance to find the hidden platform in the Morris water maze and almost 55% of the variability in the number of annulus crossings at 12 months of age (Figure 5). In contrast, there were no significant differences between control mice and those treated with Timoptic-XE when memory was assessed using a conditioned odor preference task. Drug groups did not differ significantly in short-term 24 h odor memory tests, long-term (3 month), or very long term (6 month) memory tests. However, at 12 months of age, when mice were tested for memory of rose vs. lemon odor discrimination after a 9 month interval, there was a significant negative correlation between the percentage of digging in the CS+ and visual detection performance at 12 months of age (Table 4). Thus, control mice that performed poorly in the visual detection task remembered the CS+ in this very long-term memory test better than mice that did well in the visual detection task (0.50% Timoptic-XE).

These results replicate those of our previous study with aging DBA/2J and C57BL/6J mice, as DBA/2J mice performed significantly worse than C57BL/6J mice in the vision tasks and the Morris water maze from 12 to 24 months of age but were not impaired in the conditioned odor preference task at these ages (Wong and Brown, 2007). In addition, at 18 months of age, DBA/2J mice exhibited better olfactory memory than C57BL/6J mice when tested for memory of an odor discrimination pair that they had learned when they were 12 months of age. As in the present study, visual ability was inversely correlated with long-term odor memory performance, indicating that olfactory memory improved as mice went blind (Wong and Brown, 2007).

We have found similar results when comparing visual ability with learning and memory performance in thirteen strains of mice with different visual abilities (Wong and Brown, 2006). Strain differences in visual ability accounted for a significant proportion of the variance between strains in measures of learning and memory in the Morris water maze. However, strain differences in motor learning on the Rotarod were not influenced by visual ability, and memory in the conditioned odor preference task was enhanced in mice with visual deficits (Brown and Wong, 2007). These previous studies showed that learning and memory deficits in the Morris water maze were due to strain differences in visual ability rather than strain differences in cognitive functioning. The results of the present study provide even stronger evidence that learning and memory deficits in the Morris water maze are due to visual impairment because the dissociation between visual and cognitive ability was demonstrated within individuals of the same genetically identical strain. DBA/2J mice that were blind at 12 months of age could not learn a visuo-spatial memory task that they were able to learn at 3, 6, and 9 months of age, while mice that could see at 12 months of age could learn and remember the task. Furthermore, the fact that the same mice were tested repeatedly as they aged, provides further evidence that the deficits in the Morris water maze seen in 12 month old untreated mice were due to visual impairment rather than cognitive decline (which would be most likely how the data would be interpreted if visual ability was not measured). The control mice received the same amount of previous training as treated mice but their visual dysfunction was not prevented with Timoptic-XE.

Because this was a longitudinal study and the same locations were used for place, reversal and visual platform tasks at each age in the Morris water maze, it is possible that there was some memory retention from previous training periods at 12 months of age in both treated and untreated groups. By using the same locations for place, reversal and visual platforms at each age, we were able to maximize learning performance by exploiting any residual learning that occurred over repeated testing. Furthermore, moving the platform between ages could have confounded long-term memory retention with a competing working memory procedure, as mice would not only have to learn a new platform location but also suppress any memory of the previous platform location. Although this could underestimate the acquisition deficit that would occur in 12 month animals in a cross sectional study, it helps to explain some of the non-visual “learning” that occurs in control mice at 12 months of age as these mice decreased their swim distance in the Morris water maze. We hypothesize that because control mice had previous training at 3, 6, and 9 months of age and therefore understood the rules of the task, they adopted an efficient non-visual search strategy to find the hidden platform (Janus, 2004). However, the ability to switch strategies does not indicate that all cognitive functions are intact but it does suggest that they are capable of learning a new way to find the platform when visual cues are no longer available.

It should be noted that there was some decline of spatial learning and memory ability as treated mice aged that was not completely eliminated by preventing visual loss with Timoptic-XE. This could be due to age-related cognitive decline independent of vision loss or to some visual dysfunction that was not prevented by Timoptic-XE. We think that the decline in Morris water maze performance in treated groups by 12 months of age was due to a remaining visual impairment that was not completely eliminated by Timoptic-XE rather than cognitive deficit because (1) latency and distance values in the Morris water maze were dose-dependent at 12 months of age (0.50% Timoptic-XE mice were faster and swam a shorter distance to find the hidden platform than 0.25% Timoptic-XE mice and (2) there were dose-dependent effects of Timoptic-XE in the visual system (0.50% Timoptic-XE mice had a better performance in the visual water task, lower IOP and less cell loss in the retinal ganglion cell layer than 0.25% Timoptic-XE mice) (Wong and Brown, 2012).

The results of the present study provide support for all three hypotheses of the “sensory impairment” theory. First, 12 month old visually impaired control DBA/2J mice performed poorly in a visuo-spatial learning and memory task. Second, control DBA/2J mice were not impaired when learning and memory performance was dependent on the detection of odor stimuli. Third, Timopic-XE treatment prevented visual deficits in 12 month old DBA/2J, which resulted in the preservation of visuo-spatial learning and memory ability in the Morris water maze in these mice. Similar effects on cognitive performance have been shown in humans undergoing therapy to restore visual function. For example, surgery to remove cataracts in one eye significantly improved cognitive functioning as measured by the Revised Hasegawa Dementia Scale in patients with dementia (Tamura et al., 2004) and bilateral cataract removal in visually impaired elderly patients improved cognitive scores on the Mini-Mental State Examination (Ishil et al., 2008).

Although the dissociation between visuo-spatial and odor memory tasks does not support the “common cause” hypothesis of aging, both the “sensory deprivation” and the “resource allocation” hypotheses predict that improvement of sensory function should result in an improvement of cognitive ability. However, the poor performance of 0.50% Timoptic-XE mice in the long-term odor memory task conducted at 12 months of age is inconsistent with the prediction that mice with improved visual function should also show a corresponding improvement in odor memory. Furthermore, there was not a time lag between visual and cognitive deficits in control mice, as both deficits were present at 12 months of age. Therefore, our data can only be explained by the “sensory impairment” hypothesis of aging.

In aging humans, there also appears to be a robust and consistent relationship between sensory functioning and cognitive ability, as measures of visual and auditory acuity can predict age-related differences in intellectual abilities. The Maastricht Aging study showed that changes in visual and auditory acuity predicted changes in cognitive performance after a 6 year follow-up (Valentijn et al., 2005). The Australian Longitudinal Study of Aging showed that sensory functioning explained nearly 80% of the age-related cognitive variation in verbal memory and speed (Anstey et al., 2001a) and The Berlin Aging Study demonstrated that differences in visual and auditory acuity together accounted for 93% of the age-related variance in intelligence tests covering five cognitive domains (Lindenberger and Baltes, 1997). Just as the correlations between visual ability and learning and memory in mice in the present study were not significant at younger ages, the relationship between sensory and cognitive functioning in humans is much weaker at younger ages, suggesting that the mechanisms underlying the connections between sensory and cognitive functioning are similar across the adult life span, but their expression is amplified at older ages (Baltes and Lindenberger, 1997). In order to control for age-related decrements in visual ability, Toner et al. (2012) suggested that a “vision-fair” neurological assessment be used when evaluating cognitive impairment in older individuals, including those with Alzheimer's Disease and Parkinson's disease. The use of customized stimulus arrays that control for deficits in visual contrast sensitivity can effectively compensate for age-related visual decrements and provide an accurate measure of cognitive performance in Parkinson's and Alzheimer's patients as well as normal aged control subjects (Toner et al., 2012). We also recommend the use of “vision-fair” tests and/or multiple tests that rely on other sensory abilities besides visual ability when studying cognitive functioning in aging populations of mice. Many researchers working the field of behavioral genetics are unaware of sensory deficits in their transgenic mice that are unrelated to the gene of interest, yet seriously confound their performance in tasks of higher order cognitive function, such as learning, memory or anxiety.

Although the use of visual ability differs in mice and humans, we have shown that some of the consequences are the same: visual impairment causes poor performance in cognitive tasks that rely on vision as specified by the “sensory impairment” hypothesis of aging. The ultimate value in these findings is in the implications of this work for understanding the neural basis of age-related cognitive dysfunction. For example, because both humans and animals show sensory impairment as they age, failure to understand the effects of sensory impairment could impede the progress of drug discovery platforms aimed at developing new treatments for age-related neurological disorders such as Alzheimer's Disease, Parkinson's Disease and other dementias because many of these studies use visual measures of cognitive performance to assess the efficacy of these new drug regimes. If the subjects (mouse or human) are suffering from an unrelated visual deficit and have poor performance on vision-based cognitive tests as a result, any new drugs aimed at improving cognitive performance via non-visual processes would be deemed useless.

### Conflict of interest statement

The authors declare that the research was conducted in the absence of any commercial or financial relationships that could be construed as a potential conflict of interest.
